# Integrative Analysis of Metabolomics and Transcriptomics Data Identifies Prognostic Biomarkers Associated With Oral Squamous Cell Carcinoma

**DOI:** 10.3389/fonc.2021.750794

**Published:** 2021-10-07

**Authors:** Lihua Zuo, Zhuo Chen, Lihuang Chen, Jian Kang, Yingying Shi, Liwei Liu, Shuhua Zhang, Qingquan Jia, Yi Huang, Zhi Sun

**Affiliations:** ^1^ Department of Pharmacy, The First Affiliated Hospital of Zhengzhou University, Zhengzhou, China; ^2^ Department of Oral and Maxillofacial Surgery, The First Affiliated Hospital of Zhengzhou University, Zhengzhou, China; ^3^ School and Hospital of Stomatology, Weifang Medical University, Weifang, China; ^4^ Clinical Laboratory, Chongqing Southeast Hospital, Chongqing, China; ^5^ Research and Development Department, Chongqing Huangjia Biotechnology Limited Company, Chongqing, China

**Keywords:** OSCC, metabolomics, prognostic biomarkers, succinic acid, hypoxanthine

## Abstract

**Background:**

Oral squamous cell carcinoma (OSCC) is the most malignant neoplasm in oral cancer. There is growing evidence that its progression involves altered metabolism. The current method of evaluating prognosis is very limited, and metabolomics may provide a new approach for quantitative evaluation. The aim of the study is to evaluate the use of metabolomics as prognostic markers for patients with OSCC.

**Methods:**

An analytical platform, Ultra-Performance Liquid Chromatography-Quadrupole/Orbitrap High Resolution Mass Spectrometry (UHPLC-Q-Orbitrap HRMS), was used to acquire the serum fingerprinting profiles from a total of 103 patients of OSCC before and after the operation. In total, 103 OSCC patients were assigned to either a training set (n = 73) or a test set (n = 30). The potential biomarkers and the changes of serum metabolites were profiled and correlated with the clinicopathological parameters and survival of the patients by statistical analysis. To further verify our results, we linked them to gene expression using data from the Kyoto Encyclopedia of Genes and Genomes (KEGG).

**Results:**

In total, 14 differential metabolites and five disturbed pathways were identified between the preoperative group and postoperative group. Succinic acid change-low, hypoxanthine change-high tumor grade, and tumor stage indicated a trend towards improved recurrence-free survival (RFS), whether in a training set or a test set. In addition, succinic acid change-low, hypoxanthine change-high, and tumor grade provided the highest predictive accuracy of the patients with OSCC. KEGG enrichment analysis showed that the imbalance in the amino acid and purine metabolic pathway may affect the prognosis of OSCC.

**Conclusions:**

The changes of metabolites before and after operation may be related to the prognosis of OSCC patients. UHPLC-Q-Orbitrap HRMS serum metabolomics analysis could be used to further stratify the prognosis of patients with OSCC. These results can better understand the mechanisms related to early recurrence and help develop more effective therapeutic targets.

## Introduction

Oral squamous cell carcinoma (OSCC) is the most common malignant neoplasm in oral cancer, and patients with the carcinoma had a low 5-year survival rate and poor prognosis (1–3). Although the treatment of OSCC has improved, including surgery, radiotherapy, chemotherapy, and immunotherapy (1), the current worldwide average 5-year overall survival (OS) rate is only 65% (4). Over the past decade, increasing evidence has implicated altered metabolic homeostasis to be dysregulated with OSCC malignant progression (5). The most striking feature of cancer cells is that they rewire their metabolism and nutrient acquisition patterns to meet cancer cell energy needs, and oncogene signaling pathways and OSCC metabolic activity established a strong link. Cell metabolic phenotypes can be used to predict patients’ outcomes (6). Therefore, given the feature of cancer cells, identification of more sensitive prognostic biomarkers and novel therapeutic targets are important targets of research in OSCC.

The molecular pathogenesis of OSCC is complex, which is the result of the interaction of several molecular networks (5). It involves not only the changes of specific gene and protein expressions but also a change inmetabolic processes (7). Metabolomics is an important branch of omics science, which is used to evaluate the changes of metabolites in biological samples (8, 9). Recently, a large number of metabolomics studies have focused on the exploration of disease mechanisms, the identification of potential biomarkers, the prediction of cancer prognosis, and the evaluation of treatment effect (10, 11). Most metabolomics studies of OSCC are mainly based on the metabolic profiles of saliva, serum, and tumor tissues to identify potential biomarkers for screening and early diagnosis (12). Fu et al. detected 25 amino acids in OSCC tissue by targeted metabolomics technology and proved that l-asparagine metabolism disorder mediated by asparagine synthase promoted the perineural invasion of OSCC (13). In addition, Yang et al. showed that l-glutamate, l-aspartic acid, and l-proline were identified as a group of potential diagnostic biomarkers of OSCC (14). This research group also proved that amino acid signatures are also different at different distances from the surgical margins of OSCC, which provides a new idea for determining the intraoperative safety boundary (15). However, there is no prognostic study of OSCC based on metabolomics.

In this study, ultra-high-performance liquid chromatography–quadrupole/Orbitrap high-resolution mass spectrometry (UHPLC-Q-Orbitrap HRMS) was used to acquire the serum fingerprinting profiles from a total of 103 patients of OSCC before and after the operation. The serum fingerprint information of 73 OSCC patients was used as the training set to find metabolites related to prognosis, and the serum fingerprint information of the remaining 30 OSCC patients was used as the test set to confirm the results of the training set. Transcriptome data from the Kyoto Encyclopedia of Genes and Genomes (KEGG) are also used to detect gene expression levels and find key genes and pathways related to diseases. The study was designed to uncover transcription programs, driving the observed metabolic phenotype, and a framework for future studies designed to determine how specific metabolic programs may influence the prognosis of OSCC.

## Material and Methods

### Reagents and Chemicals

The HPLC-grade methanol and acetonitrile were acquired from Fisher Scientific (Fair Lawn, NJ, USA). HPLC-grade formic acid was purchased from Aladdin Industrial Co., Ltd. (Shanghai, China). HPLC-grade water was obtained by the Millipore system (Shanghai, China). The internal standards and all the endogenous metabolite standards were acquired from J&K Scientific Ltd. (Beijing, China) and Sigma-Aldrich (St Louis, MO, USA).

### Study Design and Participant

This cross-sectional study recruited 103 patients with OSCC from The First Affiliated Hospital of Zhengzhou University who were diagnosed for the first time by oral clinicians based on the clinical criteria and postoperative pathology report (16). We excluded the patients with substance abuse, viral hepatitis, severe nephropathy, malignancies, metabolic diseases, and long-term use of estrogens, tamoxifen, or corticosteroids. In all these patients, preoperative radiotherapy or chemotherapy has not been administrated. The ethical approval for this study was obtained from the Ethical Committees of The First Affiliated Hospital of Zhengzhou University (name of IRB: Ethics Committee of Scientific Research Project of The First Affiliated Hospital of Zhengzhou University; ethical code: SB201902006). This research was conducted in accordance with the ethical guidelines of the 1975 Declaration of Helsinki.

### Treatment and Follow-Up

In the first half of the year after surgery, patients were followed up every 1 month and then every 3 months until May 2021, disease progression, death, or follow-up failure. The study was conducted at each scheduled time by patient follow-up or telephone follow-up. Progression-free survival (PFS) was selected as the endpoint and defined as the time interval from surgery to local or distant recurrence and/or metastasis, whichever occurred first. If the patient died, was lost to follow-up, or did not progress at the end of the study, the survival time was considered censored.

### Sample Collection and Preparation

At patients’ initial visit and 7 days after the operation, the venous blood of each volunteer patient was collected in the morning after overnight fasting. The blood was put into polypropylene tubes containing coagulant and cooled down in an insulated box with ice. The fresh blood samples were centrifuged at 3,000 rpm for 10 min at 4°C (Centrifuge CF16RN HITACHI, Tokyo, Japan). Then we separated and transferred supernatants (serum) into new Eppendorf tubes and immediately froze them at −80°C until analysis.

After melting on ice, the serum (100 µl of sample into 300 µl of methanol solution containing 0.05 μg/ml of l-2-chlorophenylalanine and 0.5 μg/ml of ketoprofen as internal standard) was added to the samples. After being vortexed for 1 min, the mixture was centrifuged at 13,000 pm at 4°C for 10 min, and then 200 μl of supernatant was transferred to the autosampler vial for UHPLC-MS/MS analysis.

The reproducibility and reliability of UHPLC-MS/MS system were evaluated by quality control (QC) samples. After the equipment was stabilized, six QC samples were analyzed primarily. A blank (acetonitrile) was inserted after each QC sample to wash the column. One QC sample was injected at the beginning analysis, and QC samples were evenly inserted every 10 samples in the sequence of analytical workflow.

### Ultra-High-Performance Liquid Chromatography–Quadrupole-Orbitrap Analysis

We used an UHPLC system to achieve chromatographic separation (Dionex, Themo Fisher Scientific, Waltham, MA, USA). Gradient elution was performed. Aliquots measuring five microliters from each sample were injected into an ACQUITY UHPLC^®^ BEH C_18_ (2.1 mm × 100 mm, 1.7 μm, Waters, USA). Mobile phase A was acetonitrile, and mobile phase B was water containing 0.1% formic acid. The gradient elution was as follows: 0–0.5 min, 5% A; 0.5–1.0 min, 5%–60% A; 1.0–7.0 min, 60%–80% A; 7.0–9.0 min, 80%–100% A; 9.0–11.0 min, 100% A; 11.0–11.2 min, 100%–5% A; and 11.2–13.0 min, 5% A. The flow rate was 0.2 ml/min.

The Q-Exactive Orbitrap MS was combined with the UHPLC system, which used a heated electrospray ionization (HESI) source. The mass spectra were respectively acquired in the positive and negative modes through full-mass/dd MS^2^ (data-dependent MS) scanning patterns. The instrument scanned a mass range from 80 to 1,200 *m*/*z* with a mass resolution power of 17,500 in MS/MS. The temperature of the auxiliary gas was 300°C with a flow rate of 10 arb. The ion source temperature was 350°C and the capillary temperature 320°C. The collision energy was set at 20, 40, and 60 eV with the spray voltage at 3.50 kV in the positive mode or 2.8 kV in the negative mode. The analytical sequence of every experimental sample was random.

### Identification of Differential Metabolites and Kyoto Encyclopedia of Genes and Genomes Enrichment Analysis

The comprehensive peak table (molecular weight, retention time (RT), and peak area) generated by metabolites was extracted from the raw data file using compound discoverer 3.1 software (Version 3.0, Thermo Scientific). Import the comprehensive peak table into Xcalibur™ software to realize the visualization (Version 3.0, Thermo Fisher Scientific). Then, the mass spectra and spectral data entered into Xcalibur™ software were compared with the human metabolomics database (Human Metabolome Database (HMDB), http://hmdb.ca/) and PubChem compound database to determine the different metabolites.

Obtain the genes corresponding to the differential metabolites in the HMDB, import the above genes into KEGG database for enrichment analysis, and visualize the pathways with *p <*0.05 and false discovery rate (FDR) <0.05.

### Determine the Change Multiple of Metabolites

The fold change (FC) value of each metabolite in each patient was calculated by dividing the peak area of each patient’s corresponding preoperative group by the peak area of each patient’s corresponding postoperative group, to observe the relationship between the changes of metabolites and the prognosis of patients with OSCC. Instead of the average FC value, the best cutoff value was calculated using X-Tile software (https://medicine.yale.edu/lab/rimm/research/software/) to divide the samples into metabolites change-high group and metabolites change-low group.

### Statistical Analysis

The data result set, which corresponds to the concentration of certain metabolites, contained all the *m*/*z* value, RT, and ion peak area of each sample. Principal component analysis (PCA), orthogonal partial least square discrimination analysis (OPLS-DA), variable importance in projection (VIP), and the 200 times permutation test were obtained from multivariate statistical SIMCA software (Version 14.0 Umetrics, Umea, Sweden). A Student’s t-test and FC of all the peaks were used by the SPSS 21.0 software (IBM, Chicago, IL, USA). MetaboAnalyst (https://www.metaboanalyst.ca/) was used to generate the heatmap to show the trend of change, which was created by these screened metabolites.

The 3-year recurrence-free survival (RFS) rate was evaluated using the Kaplan–Meier method and log-rank test. The Cox proportional hazards model was used to estimate the independent prognostic factors for RFS. *p*-Values <0.05 were considered statistically significant. The area under the curve (AUC) and receiver operating characteristic (ROC) curve were used to evaluate and compare the prognostic value of prognostic biomarkers.

## Results

### Clinical Characteristics of the Study Subjects

We collected a total of 73 cases with primary OSCC, including 35 males and 38 females, with a mean age of 58.5 (median 56, range 28–75) years. The mean follow-up period was 14.9 months (median = 14.2, range 2.4 to 31.8). Of these 73 patients, 39 patients were alive without recurrence, and three patients died of the disease.

The remaining 31 patients were alive but relapsed or metastasized. Due to the low number of deaths, no OS analysis was performed. The clinical parameters of all 73 patients are summarized in **Supplementary Table S1**, with all the details in the Supplementary Material.

### Metabolomics Analysis and Biomarker Identification

**Figure 1:**
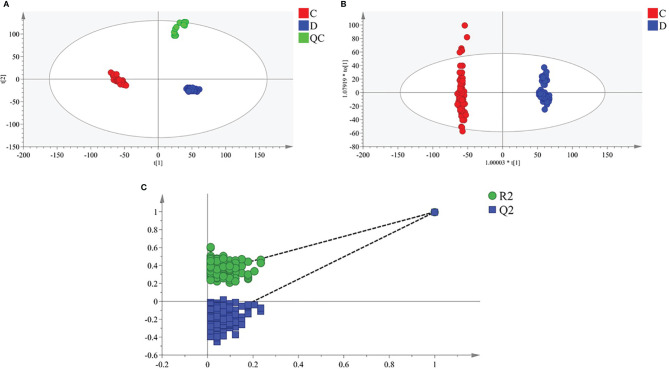
Multivariate statistical analysis of two groups. The principal component analysis (PCA) plot of QC and samples in **(A)** positive ion mode. The orthogonal partial least square discrimination analysis (OPLS-DA) score plots of preoperative group *vs.* postoperative group in **(B)** positive ion mode. Cross-validation plot with a permutation test repeated 200 times of preoperative group vs. postoperative group in **(C)** positive ion mode. C, preoperative group; D, postoperative group; QC, quality control.

**Figure 2:**
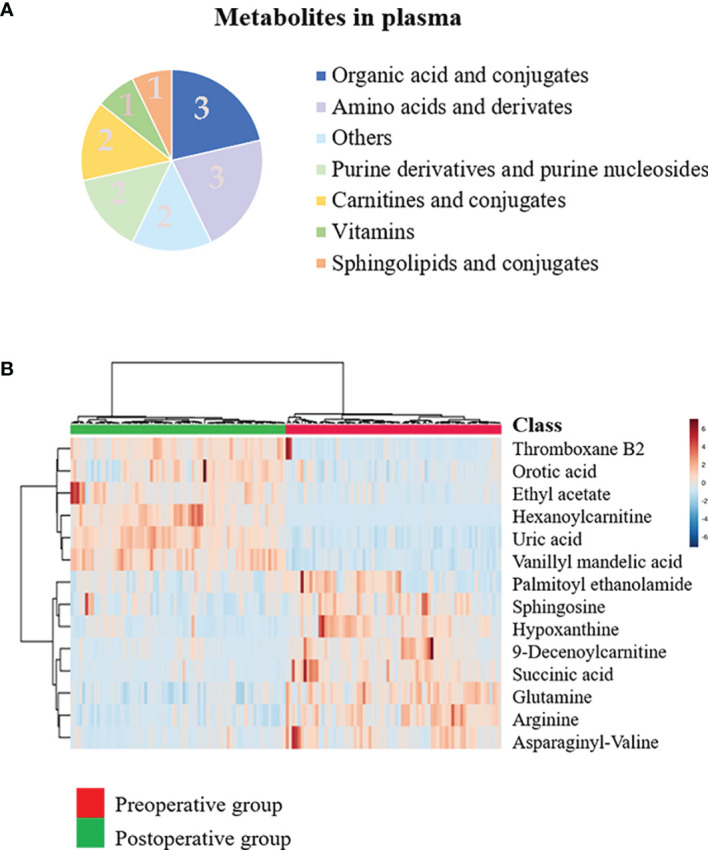
Discovery of the prognostic biomarkers for oral squamous cell carcinoma (OSCC) in plasma metabolomics. **(A)** Pie chart summarizing the taxonomic annotation of differentially expressed metabolites in the preoperative group vs. postoperative group (numbers in the pie chart represent the number of metabolites). **(B)** Heatmap representing the relative levels of 14 significantly different metabolites in the plasma samples of the preoperative group and postoperative group.

**Figure 3:**
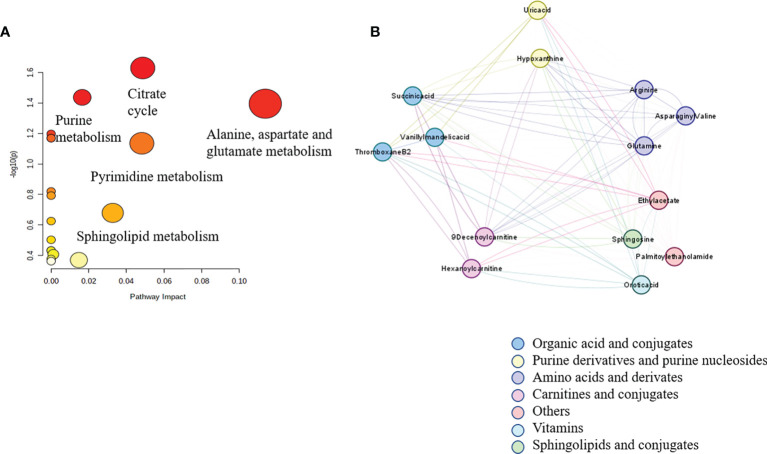
Correlation network analysis of metabolites identified in untargeted metabolomics. Spearman’s correlation analysis of 14 significantly different metabolites in the plasma samples of the preoperative group and the postoperative group in graph **(A)**. The disturbed metabolic pathways showed various metabolism changes between the preoperative group and postoperative group in graph **(B)**.

Multiple methods, including the use of internal standards and QC samples, were used to ensure stable and reliable metabolic profiling results. In PCA score plots, QC samples were clustered closely in the positive mode (**Figure 1A**), which showed that the analytical process was stable and credible. To gain insights into the metabolic features of OSCC before and after the resection, UHPLC/Q-Orbitrap HRMS was performed on these serum samples. All data of preoperative and postoperative groups were processed to normalize the ion peak areas and exported to the SIMCA 14.0 software to perform multivariate statistical analysis. A total of 2,451 ion peaks in positive ion modes were extracted. In both PCA and OPLS-DA score plots, the preoperative and postoperative groups showed a clear separation (**Figures 1A, B**), R^2^Y at 0.702 and Q^2^ at 0.553. The 200 times permutation test (**Figure 1C**) showed that the model was not over-fitting (R^2^ at 0.372 and Q^2^ at 0.997 in the posit ion mode). The results of the negative ion pattern also distinguished the preoperative group from the postoperative group in terms of metabolic changes (**Supplementary Figure S1**, Supporting Information). A combination of *p*-values <0.05 and VIP values >1 was used to identify metabolic biomarkers. In addition, a total of 14 significant metabolites (6 increased and 8 decreased in the postoperative group of patients) were annotated using online databases and reference standards, including succinic acid, hypoxanthine, glutamine, and arginine (**Table 1**). The heatmap (**Figure 2**) shows the differences in metabolite distribution between the two groups. The correlation among these 14 differentially expressed metabolites was explored using Spearman’s correlation analysis. As **Figure 3B** shows, the metabolites with smaller *p*-values were enriched in either the preoperative subjects or the postoperative groups that had stronger correlations. To further explore the underlying molecular mechanism of OSCC, the metabolic pathways of the metabolites were analyzed by MetaboAnalyst (**Figure 3A**). The results showed that citrate cycle metabolism, purine metabolism, alanine, aspartate and glutamate metabolism, pyrimidine metabolism, and sphingolipid metabolism were associated with OSCC.

**Table 1:** Statistical analysis of potential metabolic biomarkers.

No.	Metabolites	Ion mode	RT (min)	Molecular	VIP	FC (preoperative/postoperative)	*p*-Value	Class
1	Succinic acid	P	1.54	130.12	1.63	3.34	2.88E−159	OAC
2	Arginine	P	1.558	174.11	1.69	2.86	4.21E−110	AA
3	9-Decanoylcarnitine	P	1.316	313.22	1.68	2.77	7.44E−110	CC
4	Asparaginyl-Valine	P	1.143	231.122	1.63	2.59	1.51E−86	AA
5	Glutamine	P	0.968	146.07	1.62	1.83	1.48E−82	AA
6	Hypoxanthine	N	1.852	136.04	1.62	1.47	6.49E−82	PPN
7	Sphingosine	P	1.472	299.28	1.6	1.43	1.30E−80	SC
8	Palmitoyl ethanolamide	P	1.302	299.28	1.7	1.35	4.98E−78	OTH
9	Hexanoylcarnitine	P	1.725	259.18	1.61	0.64	1.10E−76	CC
10	Orotic acid	P	1.421	156.01	1.66	0.6	3.77E−74	VIT
11	Uric acid	P	9.753	168.03	1.5	0.44	4.55E−74	PPN
12	Vanillyl mandelic acid	N	1.374	198.05	1.59	0.43	1.18E−67	OAC
13	Ethyl acetate	P	1.156	88.05262	1.67	0.39	1.13E−66	OTH
14	Thromboxane B2	P	1.082	408.19712	1.5	0.39	1.29E−66	OAC

p-value, the analysis was adjusted by gender, age, BMI, smoking status, steroid use, seizure medication use, and diabetes medication use.

RT, retention time; FC, fold change; VIP, variable importance in projection, obtained from preoperative group vs. postoperative group in discovery cohort; OAC, organic acid and conjugates; AA, amino acids and derivatives; CC, carnitines and conjugates; PPN, purine derivatives and purine nucleosides; VIT, vitamins; SC, sphingolipids and conjugates; OTH, others.

### Succinic Acid Change-Low and Hypoxanthine Change-High Were Independent Prognostic Factors for 3-Year Recurrence-Free Survival

**Figure 4:**
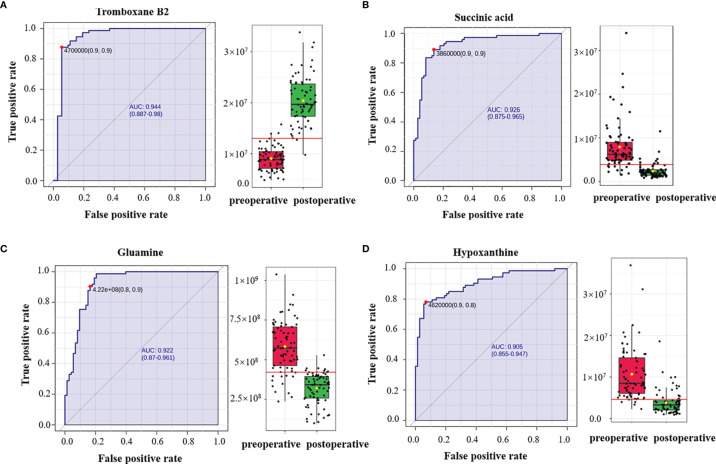
Receiver operating characteristic (ROC) curves of plasma metabolites with area under the curve (AUC) values exceeding 0.9. ROC curves of **(A)** thromboxane B2, **(B)** succinic acid, **(C)** glutamine, and **(D)** hypoxanthine. The AUCs of thromboxane B2, succinic acid, glutamine, and hypoxanthine were 0.944 (95% CI = 0.887–0.98), 0.926 (95% CI = 0.875–0.965), 0.922 (95% CI = 0.87–0.961), and 0.905 (95% CI = 0.855–0.947), respectively. The box plots show the median, the quartiles, and the whole range of peak area of these metabolites.

Of the 14 metabolites identified, ROC was performed to calculate the AUC. Four metabolites had AUC >0.9 (**Figure 4**), and ROC curves for the other four metabolites in plasma with AUC >0.8 are shown in **Supplementary Figure S2** in the Supporting Information. In the postoperative group compared with the preoperative group, the levels of succinic acid, arginine, 9-decanoylcarnitine, asparagine-valine, glutamine, hypoxanthine, sphingosine, and palmitoyl ethanolamide decreased with multiplicative changes of 3.34, 2.86, 2.77,2.59, 1.83, 1.47, 1.43, and 1.35, respectively. The levels of hexanoylcarnitine, orotic acid, uric acid, vanillyl mandelic acid, ethyl acetate, and thromboxane B2 were elevated with multiplicative changes of 0.64, 0.44, 0.6, 0.43, 0.39, and 0.39, respectively. These metabolites may provide new clues for future prognosis of OSCC.

**Figure 5:**
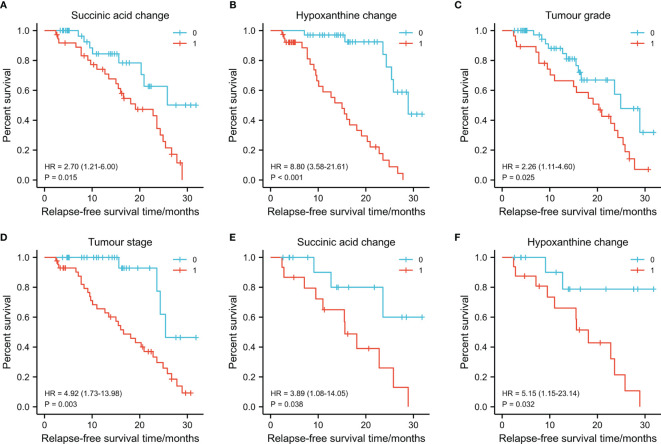
Graphs **(A–D)** showing the 3-year survival rates of succinic acid change, hypoxanthine change tumor stage, and tumor grade in 73 oral squamous cell carcinoma (OSCC) patients (training set) using the Kaplan–Meier method and log-rank test. **(A)** Succinic acid change-low had a higher 3-year recurrence-free survival (RFS) rate than succinic acid change-low (1) (79.4% vs. 30.8%, *p* = 0.015). **(B)** Hypoxanthine change-high had a higher 3-year RFS rate than hypoxanthine-low (81.6% vs. 22.9%, *p* < 0.001). **(C)** Grade I (well differentiated) had a higher 3-year RFS rate than grade II/III (moderately or poor differentiation) (53.3% vs. 17.9%, *p* = 0.025). **(D)** Early T stage (T1/T2) had a higher 3-year RFS rate than late T stage (T3/T4) (86.7% vs. 55.9%, *p* = 0.003). Graphs **(E)** and **(F)** show the 3-year survival rates of succinic acid change and hypoxanthine change in 30 OSCC patients (test set) using the Kaplan–Meier method and log-rank test. **(E)** Succinic acid change-low had a higher 3-year RFS rate than succinic acid change-low (62.4% vs. 42.8%, *p* < 0.038). **(F)** Succinic acid change-low had a higher 3-year RFS rate than hypoxanthine-low (65.6% vs. 41.6%, *p* = 0.032). 0 in graphs **(A–F)** represents succinic acid change-low, hypoxanthine change-high, grade I, early T stage (T1/T2), succinic acid change-low, and succinic acid change-low, respectively. 1 in graphs **(A–F)** represents succinic acid change-high, hypoxanthine change-low, grade II/III, late T stage (T3/T4), succinic acid change-low, and succinic acid change-high, respectively.

**Figure 6:**
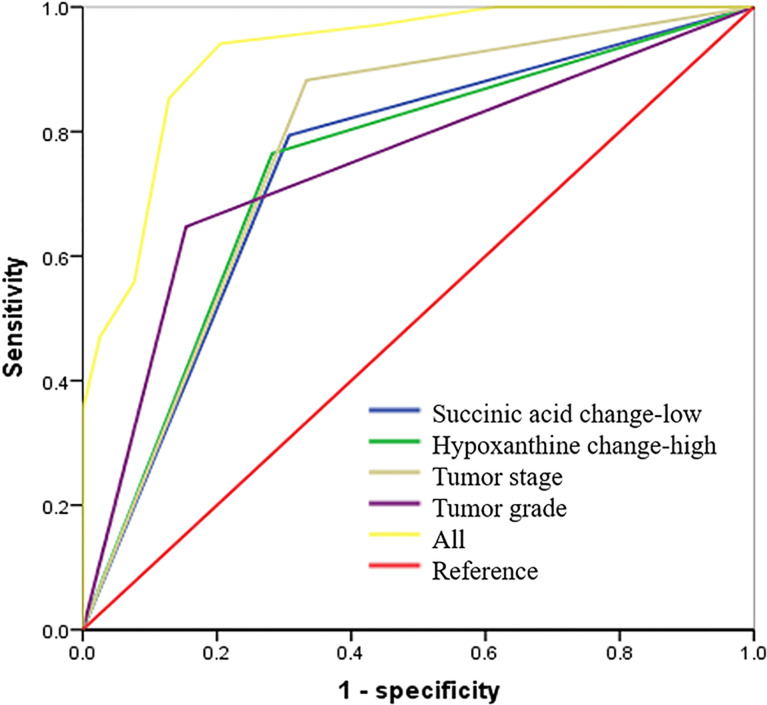
Succinic acid change-low and hypoxanthine change-high had high predictive accuracy for 3-year recurrence-free survival (RFS). receiver operating characteristic (ROC) curves indicating the predictive accuracy, sensitivity, and specificity of each potential parameter. The area under the curve (AUC) values of succinic acid change-low, hypoxanthine change-high, tumor stage, and tumor grade (differentiation) were 0.743 (95% CI = 0.627–0.859), 0.794 (95% CI = 0.686–0.903), 0.775 (95% CI = 0.664–0.885), and 0.747 (95% CI = 0.629–0.864), respectively; but succinic acid change-low combined with hypoxanthine change-high and tumor grade provided the highest predictive accuracy (AUC = 0.900; 95% CI = 0.822–0.967).

Next, we investigated the prognostic value of various clinicopathological parameters and the change of metabolites in our cohort. We divided the patients into two groups according to the cutoff value of metabolites’ FC obtained from X-Tile, with metabolites with AUC >0.8 including thromboxane B2, succinic acid, glutamine, hypoxanthine, arginine, 9-decanoylcarnitine, orotic acid, and asparaginyl-valine (**Table 2**). The univariate analysis using the log-rank test showed that tumor grade (differentiation), tumor T stage, succinic acid change-low, and hypoxanthine change-high indicated a trend towards improved RFS (**Figure 5**). Succinic acid change-low and hypoxanthine change-high were significantly associated with a better 3-year RFS rate (**Figure 6**). A total of 30 additional plasma samples in the test set were used to evaluate the potential prognostic evaluation ability of identified metabolites. Succinic acid change-low and hypoxanthine change-high are also related to the better 3-year RFS rate (**Figure 5**). Variables that showed statistically significant associations with 3-year RFS rates in the univariate analyses were entered into multivariate Cox regression analyses (**Table 2**). In multivariate analyses, succinic acid change-low expression and hypoxanthine change-high expression were independent prognostic factors for the 3-year RFS rate (hazard ratio [HR] = 5.730, 95% CI, 1.667–19. 694; [HR] = 3.221, 95% CI, 1.233–8.414).

**Table 2:** Univariate and multivariate survival analysis of clinicopathological parameters.

Variables		3-year RFS (%)	Univariate analysis (*p*)	Multivariate analysis (*p*)
**Gender**	Male	47.6	0.552	
	Female	61.3		
**Age**	<60	54.3	0.913	
	>60	52.6		
**Smoking**	Current	67.9	0.651	
	Never or former	72.6		
**Alcohol**	Current	68.1	0.727	
	Never or former	72.7		
**Tumor site**	Tongue	75.3	0.3	
	Buccal	61.5		
	Gingiva	75.7		
	Others	57.9		
**Differentiation**	Grade *I*	75.7	0.025*	0.589
	Grade II/III	61.5		
**T stage**	T1/T2	82.4	0.003*	0.384
	T3/T4	51.5		
**Succinic acid**	Low	21.4	0.015*	0.001*
	High	79.5		
**Hypoxanthine**	Low	63.3	<0.001*	0.023*
	High	80		
**Thromboxane B2**	Low	48.9	0.332	
	High	40		
**Asparaginyl-valine**	Low	44	0.582	
	High	52.2		
**Glutamine**	Low	47.6	0.552	
	High	61.3		
**Arginine**	Low	54.5	0.198	
	High	34.5		
**9-Decanoylcarnitine**	Low	43.1	0.723	
	High	54.5		
**Orotic acid**	Low	35.4	0.24	
	High	52		

Variables that showed statistically significant associations with 3-year RFS rates in the univariate analyses were entered into multivariate Cox regression analyses.

RFS, recurrence-free survival.

^*^p < 0.05.

### Metabolic Prognostic Panel Had High Predictive Accuracy on 3-Year Recurrence-Free Survival

To determine the predictive accuracy of succinic acid change-low and hypoxanthine change-high on 3-year RFS, we performed ROC curve analyses. As shown in **Figure 6** and **Table 3**, T-stage, tumor differentiation, succinic acid change-low, and hypoxanthine change-high had similar predictive accuracies (*p* < 0.05), but the panel of succinic acid change-low, hypoxanthine change-high, and tumor grade (differentiation) provided the highest predictive accuracy (AUC = 0.730; 95% CI, 0.654–0.805).

**Table 3:** Summary of the RFS predictive accuracy of succinic acid and hypoxanthine.

Predictive factors	AUC	SE	*p*-Value	95% CI	
T stage	0.775	0.056	<0.000*	0.664	0.885
Succinic acid change-low	0.641	0.059	<0.000*	0.627	0.859
Hypoxanthine change-high	0.682	0.055	<0.000*	0.686	0.903
Differentiation	0.656	0.06	<0.000*	0.629	0.864
All^a^	0.9	0.034	<0.000*	0.822	0.967

Note. SE, standard error; 95% CI, 95% confidence interval; RFS, recurrence-free survival; AUC, area under the curve.

a Succinic acid change-low, hypoxanthine change-high, and differentiation.

*p < 0.05.

### Biological Networks Associated With the Differentially Expressed Metabolites and Genes

**Figure 7:**
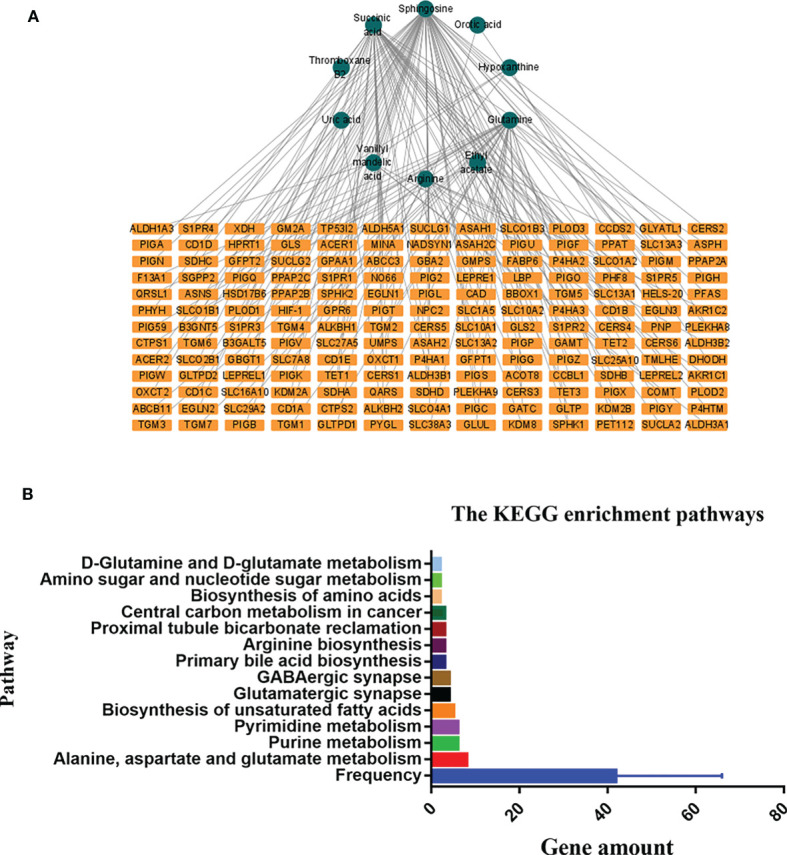
The connected network of metabolites and genes in graph **(A)**. The orange rectangles circles node represents the differentially expressed metabolites in the preoperative group vs. postoperative group. The blue rectangles represent the genes closely correlated with those metabolites. The Kyoto Encyclopedia of Genes and Genomes (KEGG) enrichment pathways in the preoperative group and postoperative group in graph **(B)**.

The HMDB and KEGG database revealed some genes that are closely correlated with the 14 differentially expressed metabolites. The “metabolites-genes” network (**Figure 7A**) was then constructed by using Cytoscape software 3.8.2. However, no related genes were found for palmitoyl ethanolamide, hexanoylcarnitine, asparaginyl-valine, and 9-decanoylcarnitine.

**Figure 8:**
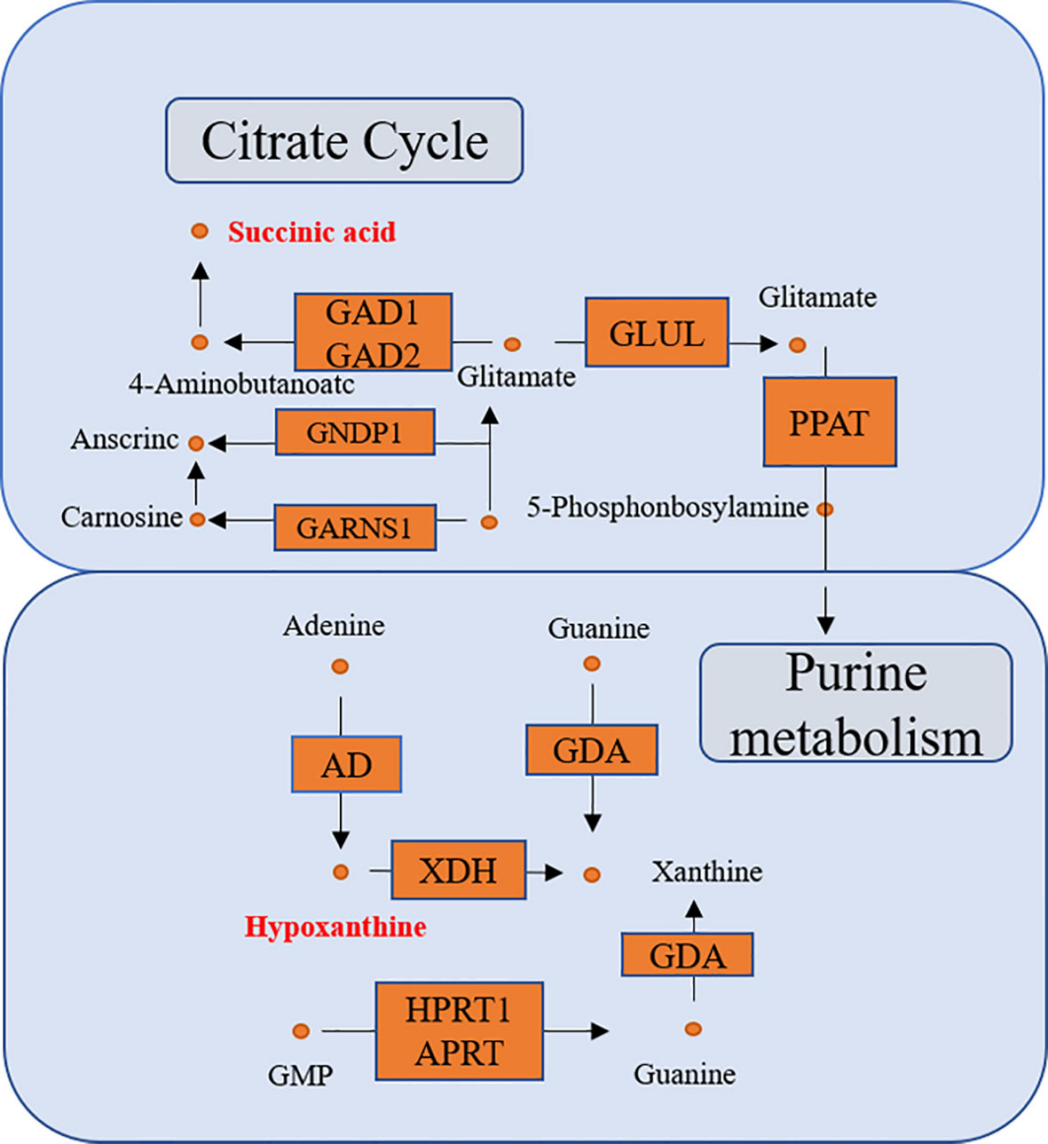
The connected network of genes, enzymes, and metabolites. The orange rectangles and circles represent the significant genes and metabolites, respectively.

KEGG enrichment analysis showed that a total of 13 pathways (*p* < 0.05, FDR < 0.05) were significantly disturbed in the postoperative group compared with the preoperative group (**Figure 7B**). The most important genes are involved in amino acid metabolism and purine metabolism. In order to further explore the relationship between genes and metabolism, the changed metabolites and genes were mapped to the relevant networks by searching the online KEGG databases and HMDB. The metabolic profiles of succinic acid and its regulatory genes and the metabolites of hypoxanthine and its regulatory genes are shown in **Figure 8**. These results suggest that a considerable number of genes in amino acid metabolism and purine metabolism pathway are closely related to the prognosis of OSCC patients.

## Discussion

As one of the major components of systems biology, metabolomics is a well-established method to assess global metabolic profiles through biomarker discovery in accessible biofluids (15, 17, 18). In this study, global non-targeted metabolomics was established to investigate changes in metabolic phenotypes associated with OSCC, and transcriptome analysis was performed to reveal genes associated with metabolites found to be differentially expressed in OSCC patients. This suggests that several metabolites and genes are commonly involved in metabolic pathways and regulatory signaling in OSCC. As a series of works, our study not only dissects the regulatory features of metabolic networks in OSCC but also explores their ability to predict prognosis in OSCC.

Low succinate in OSCC is associated with better 3-year RFS of the patients, suggesting that succinate accumulation is associated with a worse prognosis. Succinate is an inhibitor of prolyl hydroxylase (PHD) (19), which is responsible for hydroxylation of hypoxia-inducible factor 1-alpha (HIF1α), causing its degradation. Then, succinate accumulation results in a pseudo-hypoxic response that is caused by HIF1α stabilization and activation of genes containing HIF response elements (HREs) (20–22). Overall, succinate accumulation plays an important role in the epigenetic alteration of cancer cells, cancer cell metabolism, epithelial-to-mesenchymal transition (EMT), and angiogenesis.

Succinate accumulation induces epigenetic alterations in cancer cells, which causes competitive inhibition of several alpha-ketoglutarate (αKG)-dependent dioxygenase. In these αKGs, Ten-eleven-translocation (TET) and Jumonji domain-containing histone demethylases (JMHD) are responsible for histone hypermethylation and decrease of hydroxylation of 5mC (20, 23). JMHD causes the oxidation of methyl groups on lysine residues of histones H3 and H4. Its inhibition induces global histone hypermethylation that alters epigenetic control of gene expression, with potential tumorigenic consequences (3, 24). The effect of succinic acid accumulation on cell transcriptome mediates the pseudo-hypoxic phenotype and induces the change of metabolic phenotype, which leads to the bioenergy conversion from mitochondrial respiration to cytosolic glycolysis (22, 25, 26). The accumulation of succinic acid can lead to the loss of succinate dehydrogenase (SDH) activity and lead to changes in the metabolism of non-essential amino acids, especially aspartic acid, which is the main precursor of protein and nucleotide biosynthesis, as well as other non-essential amino acids such as arginine and asparagine (27). Hypermethylation induced by succinic acid accumulation promotes EMT, migration, and invasion (27, 28).

EMT allows epithelial cancer cells to present mesenchymal features, providing them with enhanced motility and invasiveness, thus allowing cancer to spread and metastasize. Hypermethylation induced by succinic acid accumulation promotes EMT, migration, and invasion (28). Succinate accumulation also promotes angiogenesis. In SDH deficient prostaglandins and prostate cancer tissues, learner found that succinic acid accumulation was associated with expression of inducible factor-1 α, angiogenic genes and high density of microvessels. (19, 29, 30).

In the past, succinic acid was considered as an intermediate of citric acid cycle. However, it also plays a role in gene expression and intercellular communication (10). Recently, the importance of succinic acid accumulation in carcinogenesis progression has been fully demonstrated, which fully proves that succinic acid is a tumor-related metabolite. Serum organic acid analysis can be used as an effective and cheap broad-spectrum screening method to narrow the scope of more expensive gene sequencing (31).

In our study, hypoxanthine change-high is associated with better 3-year RFS, suggesting that consumption of hypoxanthine is associated with a worse prognosis. Hypoxanthine-guanine phosphoribosyl transferase (HPRT) is an enzyme in the DNA salvage pathway responsible for recycling GTP and is involved in the production and regulation of the purinosome, with a significant regulatory role in the synthesis rate of purines during the cell cycle. It is significantly elevated in cancer cells (32, 33). Hypoxanthine is one of the substrates of HPRT. With the increase of HPRT level, the consumption of hypoxanthine is more. Wang et al. reported that HPRT promotes proliferation and metastasis in head and neck squamous cell carcinoma, which concurs with the results in the present study (34).

The discovery and detection of metabolites in serum of patients with cancer have created a new paradigm of cancer biology. It is possible to detect metabolites related to early prognosis and take corresponding treatment. This includes the discovery of new therapeutic targets that exploit vulnerabilities of cancer cells, such as their dependence on oncometabolites. Measurement of succinic acid, hypoxanthine, and other metabolites will be an ideal tool for screening and tracking OSCC with corresponding metabolic disorders (35). Advances in MS and nuclear magnetic resonance technology have promoted high-resolution metabolite mapping of cells and tumors and have identified the accumulation of metabolites associated with specific gene defects (36).

In conclusion, this study established a new method to evaluate the prognosis of patients with OSCC, using UHPLC-Q-Orbitrap HRMS serum metabolomics analysis, which showed higher predictive accuracy in patients with OSCC. However, it is not so accurate to only use FC mean values as a standard. In the future, more samples need to be collected to standardize the measurement.

## Data Availability Statement

The original contributions presented in the study are included in the article/**Supplementary Material**. Further inquiries can be directed to the corresponding author.

## Ethics Statement

The studies involving human participants were reviewed and approved by the Ethical Committees of The First Affiliated Hospital of Zhengzhou University (name of IRB: Ethics Committee of Scientific Research Project of The First Affiliated Hospital of Zhengzhou University; ethical code: SB201902006). The patients/participants provided their written informed consent to participate in this study.

## Author Contributions

ZS designed the study. LZ assisted in the conceptualization of the study. ZC and QJ conducted the experiments. ZC and LC undertook the data analysis and wrote the manuscript. SZ and YH collected the assay samples. JK, YS, and LL aided in editing the manuscript. All authors contributed to the article and approved the submitted version.

## Funding

This work was supported by the National Natural Science Foundation of China (No. 82003921), the Foundation of Beijing medical and health (No. YWJKJJHKYJJ-B17818), the key scientific research project of Henan institution of higher education (No. 19A360008 and No. 21A320057), the Joint Construction Project of Henan Medical Science Project (No. SBGJ22002078), and the Health Science and Technology Innovation Excellent Talents Training Project for Young and Middle-aged in Henan Province (No. YXKC2020058).

## Conflict of Interest

Author YH was employed by Chongqing Huangjia Biotechnology Limited Company.

The remaining authors declare that the research was conducted in the absence of any commercial or financial relationships that could be construed as a potential conflict of interest.

## Publisher’s Note

All claims expressed in this article are solely those of the authors and do not necessarily represent those of their affiliated organizations, or those of the publisher, the editors and the reviewers. Any product that may be evaluated in this article, or claim that may be made by its manufacturer, is not guaranteed or endorsed by the publisher.

## Supplementary Material

Supplementary Tables S1 The baseline characteristics of 73 participants

Supplementary Figures S1 Multivariate statistical analysis of two groups. The principal component analysis (PCA) plot of QC and samples in **(A)** the negative positive ion mode. The orthogonal partial least square discrimination analysis (OPLS-DA) score plots of preoperative group vs. postoperative group in **(B)** negative ion mode. Cross-validation plot with a permutation test repeated 200 times of preoperative group vs. postoperative group in **(C)** negative ion mode. C, preoperative group; D, postoperative group; QC, quality control.

Supplementary Figures S2 ROC curves of plasma metabolites with AUC values exceeding 0.8 in our study.

Supplementary Figures S3 The complete analyses steps of a flowchart in our study.
