# Supplementary material for: Integrative Analysis of Metabolomics and Transcriptomics Data Identifies Prognostic Biomarkers Associated With Oral Squamous Cell Carcinoma
Source: Front Oncol. 2021 Oct 7;11:750794. doi: 10.3389/fonc.2021.750794 (PMC8529182; doi:10.3389/fonc.2021.750794)
Supplement: Supplementary Tables S1 — The baseline characteristics of 73 participants [file Table_1.docx]

**Table 1 The baseline characteristics of 73 participants**

| **Clinical characteristic** | **Category** | **N%** |
| --- | --- | --- |
| **Gender** | male | 42(57.5) |
|  | female | 31(42.5) |
| **Age** | >65 | 35(47.9) |
|  | <65 | 38(52.1) |
| **Smoking** | Current | 36(49.3) |
|  | Never or Former | 37(50.1) |
| **Alcohol** | Current | 39(53.4) |
|  | Never or Former | 34(46.6) |
| **Tumour site** | Tongue | 29(39.7) |
|  | Bucca | 19(26.0) |
|  | Gingiva | 18(24.7) |
|  | Others | 10(13.7) |
| **Differentiation** | GradeⅠ | 38(52.1) |
|  | GradeⅡ/Ⅲ | 35(47.9) |
| **T stage** | T1/T2 | 30(41.1) |
|  | T3/T4 | 43(58.9) |
| **Nodal invasion** | Negative | 29(39.7) |
|  | Positive | 44(60.3) |
| **Succinic acid** | Low | 39(53.4) |
|  | High | 34(46.6) |
| **Hypoxanthine** | Low | 38(52.1) |
|  | High | 35(47.9) |
| **Thromboxane B2** | Low | 49(67.1) |
|  | High | 25(34.3) |
| **Asparaginyl-valine** | Low | 50(68.5) |
|  | High | 23(31.5) |
| **Glutamine** | Low | 44(60.3) |
|  | High | 29(39.7) |
| **Arginine** | Low | 51(69.9) |
|  | High | 22(30.1) |
| **9-Decenoylcarnitine** | Low | 48(65.8) |
|  | High | 25(34.2) |
| **Orotic acid** | Low | 42(57.5) |
|  | High | 31(42.5) |
