# Supplementary figures and images for: Integrative Analysis of Metabolomics and Transcriptomics Data Identifies Prognostic Biomarkers Associated With Oral Squamous Cell Carcinoma
Source: Front Oncol. 2021 Oct 7;11:750794. doi: 10.3389/fonc.2021.750794 (PMC8529182; doi:10.3389/fonc.2021.750794)

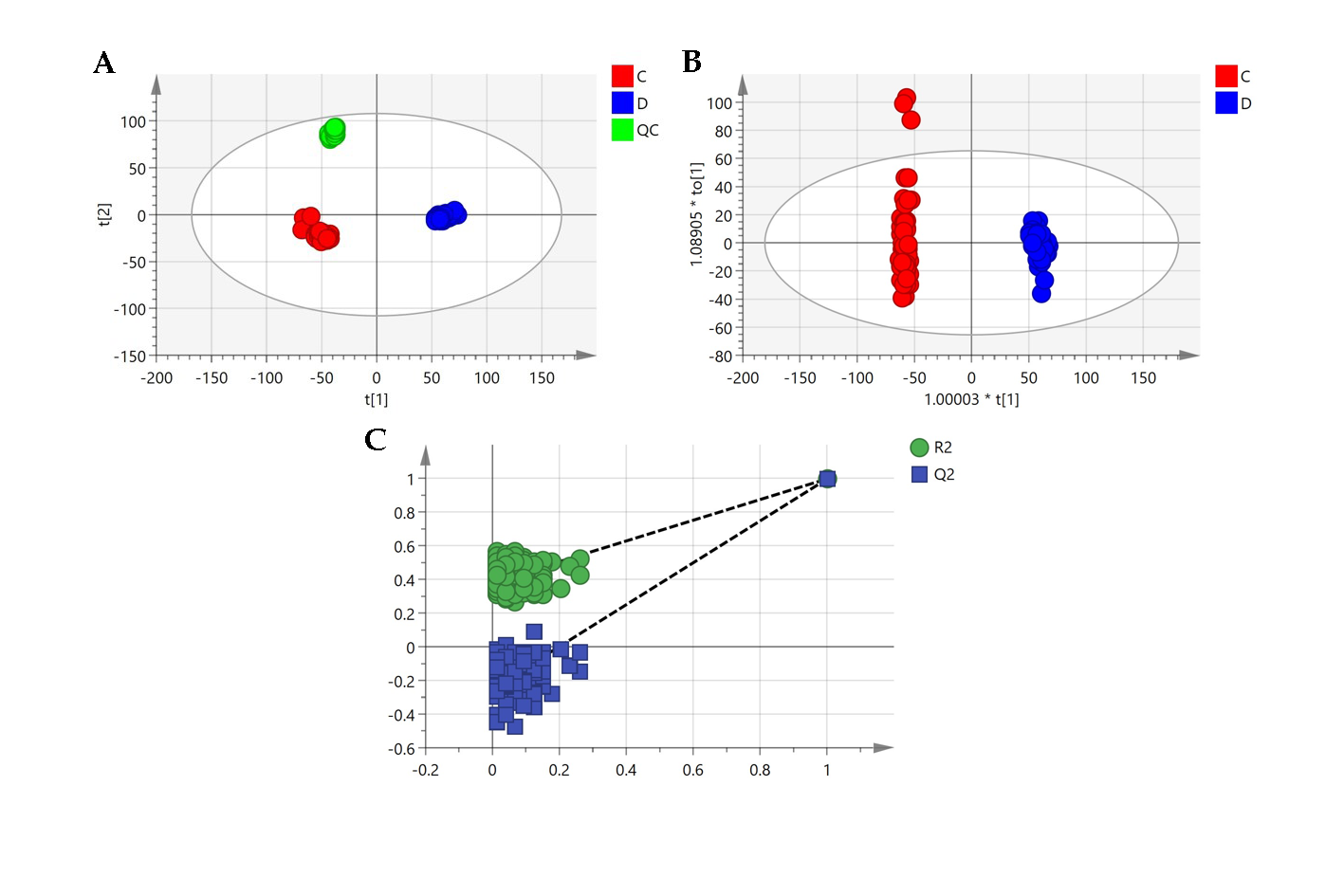

Supplement: Supplementary Figures S1 — Multivariate statistical analysis of two groups. The principal component analysis (PCA) plot of QC and samples in (A) the negative positive ion mode. The orthogonal partial least square discrimination analysis (OPLS-DA) score plots of preoperative group vs. postoperative group in (B) negative ion mode. Cross-validation plot with a permutation test repeated 200 times of preoperative group vs. postoperative group in (C) negative ion mode. C, preoperative group; D, postoperative group; QC, quality control. [file Image_1.tif]

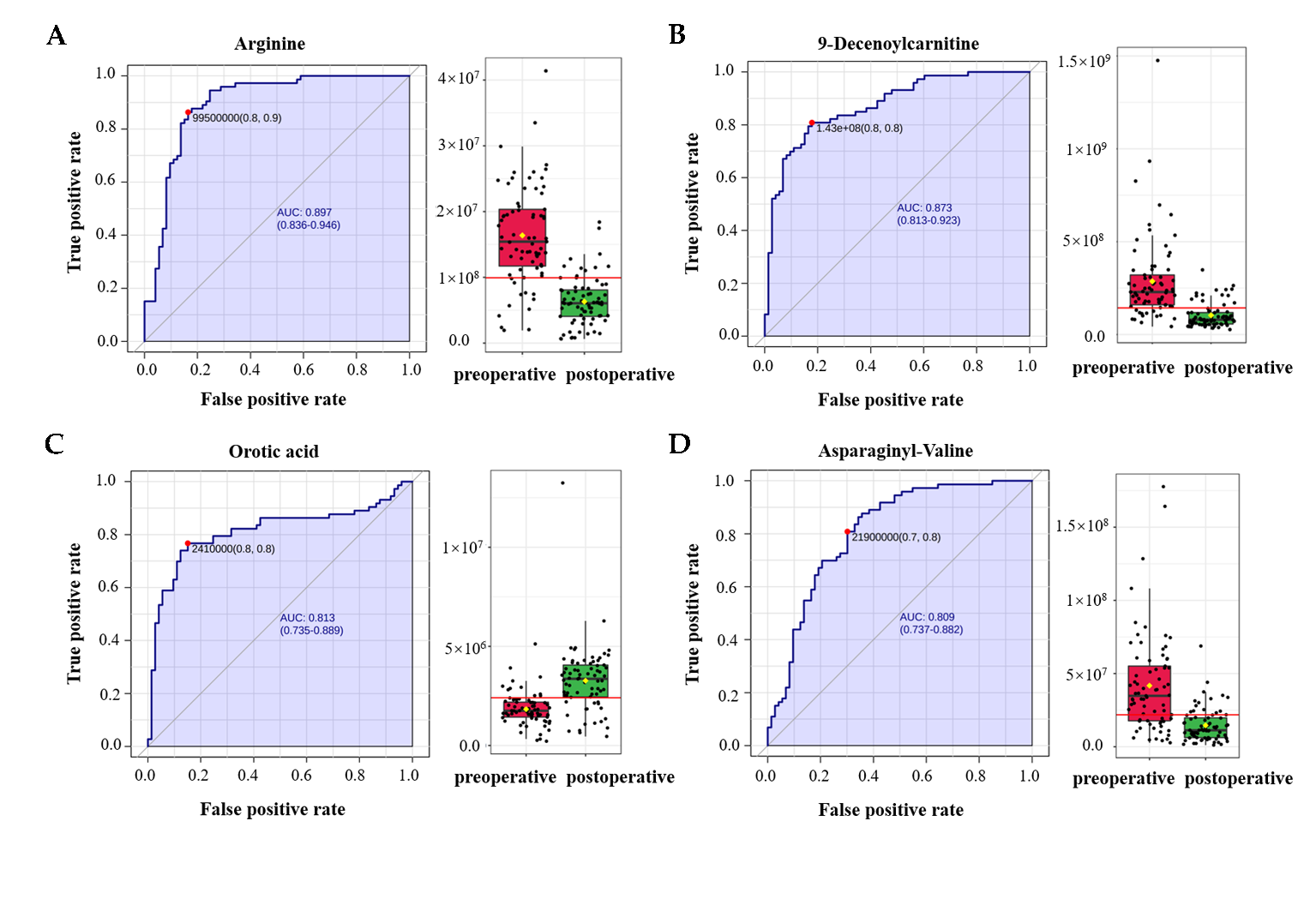

Supplement: Supplementary Figures S2 — ROC curves of plasma metabolites with AUC values exceeding 0.8 in our study. [file Image_2.tif]

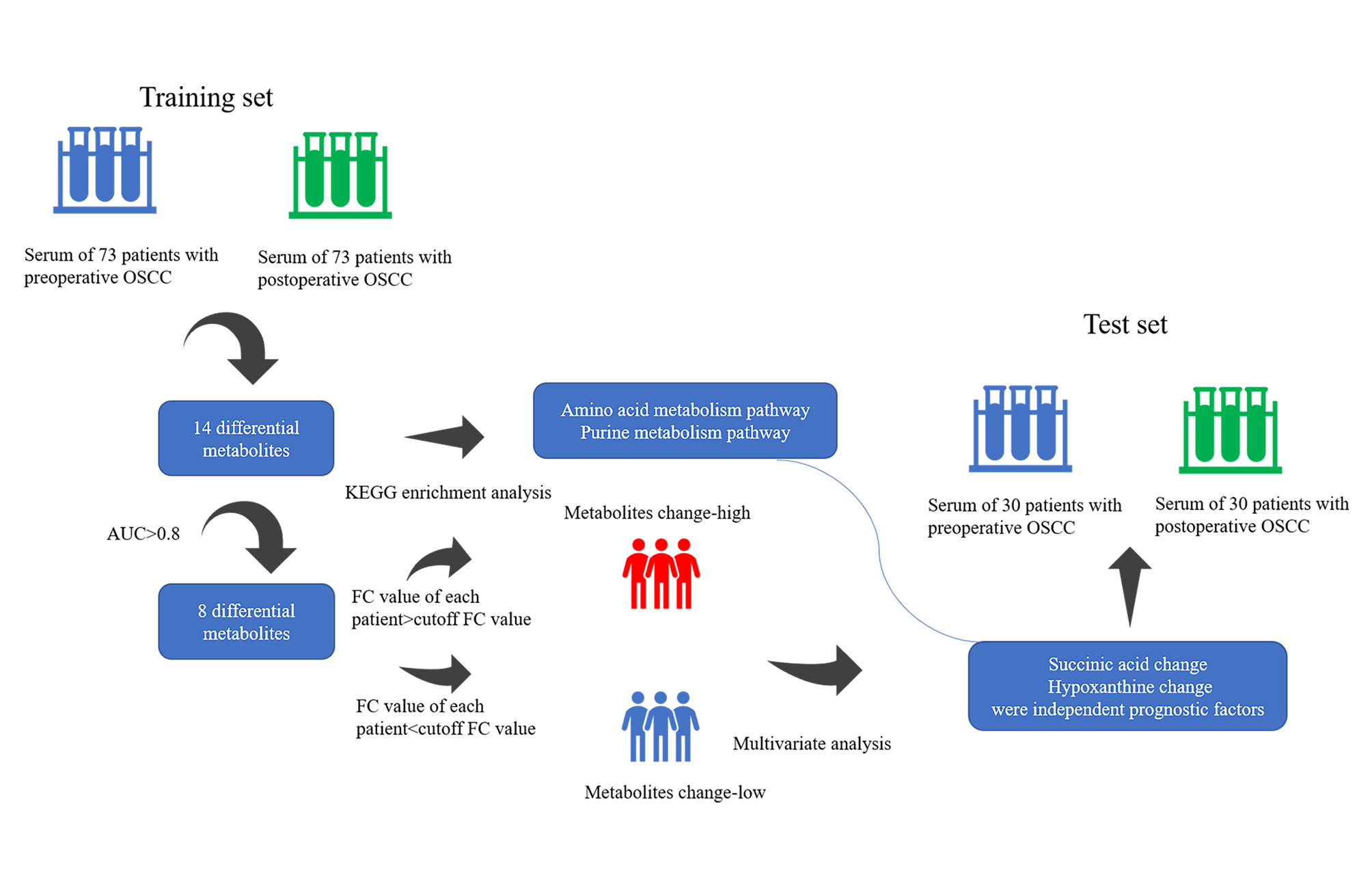

Supplement: Supplementary Figures S3 — The complete analyses steps of a flowchart in our study. [file Image_3.tif]
